# Non‐nucleoside reverse transcriptase inhibitor levels among HIV‐exposed uninfected infants at the time of HIV PCR testing – findings from a tertiary healthcare facility in Pretoria, South Africa

**DOI:** 10.1002/jia2.25284

**Published:** 2019-06-19

**Authors:** Ahmad Haeri Mazanderani, Tanya Y Murray, Gayle G Sherman, Tracy Snyman, Jaya George, Theunis Avenant, Ameena E Goga, Michael S Pepper, Nicolette du Plessis

**Affiliations:** ^1^ Centre for HIV & STIs National Institute for Communicable Diseases National Health Laboratory Service Johannesburg South Africa; ^2^ Department of Medical Virology Faculty of Health Sciences University of Pretoria Pretoria South Africa; ^3^ Paediatric HIV Diagnostics Wits Health Consortium Johannesburg South Africa; ^4^ Department of Paediatrics & Child Health Faculty of Health Sciences University of the Witwatersrand Johannesburg South Africa; ^5^ Department of Chemical Pathology National Health Laboratory Service and University of Witwatersrand Johannesburg South Africa; ^6^ Paediatric Infectious Diseases Division Department of Paediatrics Kalafong Provincial Tertiary Hospital Pretoria South Africa; ^7^ Department of Paediatrics and Child Health Faculty of Health Sciences University of Pretoria Pretoria South Africa; ^8^ Health Systems Research Unit South African Medical Research Council Cape Town South Africa; ^9^ Institute for Cellular and Molecular Medicine Department of Immunology SAMRC Extramural Unit for Stem Cell Research and Therapy Faculty of Health Sciences University of Pretoria Pretoria South Africa

**Keywords:** PMTCT, option B+, antiretrovirals, efavirenz, nevirapine, toxicity, adherence, early infant diagnosis

## Abstract

**Introduction:**

To date, very little programmatic data has been published regarding serial antiretroviral (ARV) levels in infants exposed to maternal treatment and/or infant prophylaxis during the first months of life. Such data provide the opportunity to describe the proportion of infants exposed to virologically suppressive levels of ARVs and to gauge adherence to the prevention of mother‐to‐child transmission of HIV (PMTCT) programme.

**Methods:**

From August 2014 to January 2016, HIV‐exposed infants born at Kalafong Provincial Tertiary Hospital in Pretoria, South Africa were enrolled as part of an observational cohort study. Plasma samples from HIV‐exposed uninfected infants were obtained at birth, 6‐weeks, 10‐weeks and 14‐weeks of age and quantitative efavirenz (EFV) and nevirapine (NVP) drug level testing performed using liquid chromatography‐mass spectrometry, irrespective of maternal ARV regimen. Descriptive analysis of EFV and NVP levels in relation to self‐reported maternal and infant ARV exposure was performed. EFV levels >500 ng/mL and NVP levels >100 ng/mL were reported based on studies suggesting that trough levels above these thresholds are associated with virological suppression and PMTCT respectively.

**Results:**

Among 66 infants exposed to maternal EFV *in utero*, 29 (44%) had virologically suppressive plasma EFV levels at birth, with a median level of 1665 ng/mL (IQR: 1094 to 3673). Among infants who were exclusively breastfed at 6‐, 10‐ and 14 weeks, 13/48 (27%), 5/25 (25%) and 0/21 (0%) had virologically suppressive EFV levels. Among 64 infants whose mothers reported administering daily infant NVP at time of their 6‐week HIV PCR test, only 45 (70%) had NVP levels above the minimum prophylactic trough level.

**Conclusions:**

During the first 10‐weeks after delivery, a quarter of breastfed infants born to women on an EFV‐containing treatment regimen maintained virologically suppressive EFV plasma levels. This finding highlights the importance of both careful monitoring of ARV side effects and repeat HIV PCR after the first few months of life among HIV‐exposed uninfected infants. As 30% of infants had inadequate NVP plasma levels at 6‐weeks of age, adherence counselling to caregivers regarding infant prophylaxis needs to be enhanced to further reduce mother‐to‐child transmission of HIV.

## 1 Introduction

As combination antiretroviral therapy (cART) becomes more readily available in resource‐limited settings, a rapid rise in the number of foetal antiretroviral (ARV) drug exposures is expected 1. Current World Health Organization (WHO) guidelines for the prevention of mother‐to‐child transmission of HIV (PMTCT) recommend that all pregnant and breastfeeding women living with HIV (WLHIV) be initiated on life‐long cART and all breastfed HIV‐exposed infants receive daily antiretroviral prophylaxis such as nevirapine (NVP) syrup for at least six weeks (Option B+) 2. Hence, infants are being exposed to a number of different ARV drugs over a long duration. This includes *in utero* exposure to maternal ARVs and postnatal exposure to both prophylactic regimens and maternal ARVs transferred in breastmilk. Changes in breastfeeding policy, with the WHO recommending that WLHIV continue to breastfeed for a two‐year duration under cART cover, are likely to prolong ARV exposure even further among HIV‐exposed infants 3. Whereas there are clear benefits of therapeutic and prophylactic ARV regimens for maternal health and prevention of perinatal HIV acquisition 4, 5, the clinical implications of ARV exposure among infants remain underdetermined 1.

HIV‐exposed uninfected (HEU) infants have substantially higher morbidity and mortality compared with HIV‐unexposed infants 6. Many reasons and mechanisms are likely to account for these differences, including social determinants of health, immune activation, and infant ARV exposure 7. Regarding the latter, women who start cART prior to conception, in comparison to those who initiate cART after conception, are more likely to deliver preterm, very preterm, or low‐birthweight infants 8, 9. *In utero* cART exposure has also been associated with significantly lower length‐for‐age and weight‐for‐age at 24 months among HEU infants 10. Importantly, preterm delivery and suboptimal infant growth are associated with significant infant morbidity and mortality in resource‐limited settings 11, 12. Furthermore, HEU children may be at increased risk of cognitive and motor delays, possibly related to ARV exposure, although there is conflicting data regarding these observations 13. The associations between exposure to nucleoside reverse transcriptase inhibitors (NRTI), mitochondrial toxicity and neurodevelopment have been investigated, with equivocal results. However, there is mounting concern that *in utero* exposure to efavirenz (EFV), a non‐nucleoside reverse transcriptase inhibitor (NNRTI), could have serious neurodevelopmental and neuropsychiatric consequences, with very few studies having evaluated this phenomenon thus far 6, 14.

In addition to the pharmacodynamic aspects of ARV exposure and infant health, there is concern that ARV prophylaxis may impact negatively on the sensitivity of virological assays 15. The performance of virological assays in the context of PMTCT is critical to inform infant testing algorithms. The WHO recommends that early infant diagnosis (EID) tests have a sensitivity of at least >95% (preferably >98%) and a specificity of >98% 2. Although numerous EID assays meet these criteria and have been approved for *in vitro* diagnostic use, validation studies typically do not assess diagnostic sensitivity among infants exposed to ARVs. Suboptimal sensitivity of EID assays among infants exposed to various prophylactic regimens has increasingly been reported, including sensitivity of approximately 89% at one month of age among infants given daily zidovudine (AZT) prophylaxis and 80% at two months of age among infants exposed to triple‐drug prophylaxis 16, 17.

To date, very little programmatic data has been published regarding serial ARV levels in infants exposed to maternal cART and/or infant ARV prophylaxis. Such data provides the opportunity to describe the proportion of infants exposed to potentially toxic levels of ARVs, to anticipate the potential for infant virological suppression should the infant be infected (and thus poor performance of infant diagnostic tests), and to gauge adherence to the PMTCT programme. This paper describes infant EFV and NVP plasma levels at birth, 6‐, 10‐ and 14‐weeks postdelivery to understand the interplay between maternal EFV‐use and infant EFV levels, and infant NVP prophylaxis adherence.

## 2 Methods

### 2.1 Setting

From August 2014 to January 2016, samples were obtained from a prospective observational cohort of HEU infants participating in the Very Early Infant Diagnosis (VEID) study at Kalafong Provincial Tertiary Hospital (KPTH), an academic facility situated in Pretoria, South Africa. Infants were enrolled at birth, defined as <72 hours after delivery. Infant blood samples for HIV PCR testing were collected at birth, 6‐, 10‐ and 14‐weeks of age. Clinical examination was performed at each time‐point by a qualified medical doctor. During the study period all WLHIV, irrespective of CD4 count or clinical stage, were eligible for cART during pregnancy and breastfeeding. As per national guidelines, first line cART regimens for adults comprised a triple drug combination of tenofovir, lamivudine/emtricitabine, and EFV. For patients with a contraindication to EFV, such as active psychiatric illness, NVP was recommended instead (if CD4 <250 cells/μL) 18. All HIV‐exposed infants were eligible for at least a 6‐week duration of daily NVP prophylaxis, with high‐risk infants provided with either dual AZT/NVP prophylaxis for 6‐weeks or extended duration daily NVP for 12‐weeks 18. Prophylactic NVP doses were prescribed according to the national guidelines: 15 mg/day for infants >2.5 kg, 10 mg/day for infants 2.0 to 2.5 kg, and 2 mg/kg for the first two weeks followed by 4 mg/kg for the next four weeks for infants <2.0 kg 18.

Whole blood EDTA specimens were taken at each time point for HIV PCR testing. After HIV PCR testing, the remaining whole blood specimen was spotted on a filter‐paper dried blood spot card (3 to 5 spots per card; 70 μL per spot) as per standard laboratory procedure. Any left‐over specimen was centrifuged and plasma was stored at −70°C.

All infants included in this substudy tested HIV PCR negative at birth, 6‐, 10‐ and 14‐weeks of age. Furthermore, no signs of drug‐toxicity were observed on routine clinical examination at these time points.

### 2.2 Antiretroviral exposure variables

Data regarding maternal cART exposure, regimen and infant feeding were obtained during interviews with enrolled participants at each study visit using standardized questionnaires. Data regarding age at first NVP dose and age (in hours) of blood sampling at birth were obtained from clinical records, with time from first NVP dose to blood sampling calculated for each participant. All infants were discharged with a supply of NVP syrup to last for at least a 6‐week duration. Data regarding NVP prophylaxis use at subsequent visits was obtained during interviews with enrolled participants at the respective time points.

Regarding clinically significant NNRTI levels, EFV has been found to have a higher potency than NVP with an *in vitro* protein‐adjusted 95% inhibitory concentration (IC_95_) for HIV‐1 wild‐type virus of 8 versus 190 ng/mL respectively 19. An EFV plasma mid‐dosing and trough concentration target of 1000 to 4000 ng/mL is usually cited, with levels of >4000 ng/mL being associated with increased risk of side‐effects 20, 21. These data are, however, derived from adult clinical monitoring studies with data from children suggesting that an increased risk of viral replication occurs at a much lower trough level of <650 ng/mL 20, 22. Regarding NVP, target trough levels required for prophylaxis are lower than those proposed for treatment. Whereas a therapeutic trough level of >3000 ng/mL has been described for adult patients (a therapeutic trough level target has not been defined for infants), a prophylactic trough target of >100 ng/mL (10 times the IC_50_) is usually cited for infants during the period of HIV exposure 23.

### 2.3 Laboratory methods to assess ARV levels

Quantitative plasma EFV and NVP drug level testing was performed on all samples, irrespective of maternal cART regimen, using liquid chromatography‐mass spectrometry (Shimadzu 8060). Matrix matched standards and controls (Chromsystems, Munich, DE) were used to create a 7‐point standard curve. Samples were thawed to room temperature. A volume of 5 μL of deuterated internal standard was added to 25 μL of plasma which was then extracted with 200 μL of acetonitrile for protein precipitation. Samples were vortexed vigorously and then centrifuged for 10 minutes at 14000 RPM; 2 μL of supernatant was injected onto an Acquity T3 Column (Waters, Massachusetts, USA) with a total run time of 5.5 minutes per sample. Efavirenz and NVP were analysed using a mobile phase composition of deionised water with 0.1% formic acid (A) and acetonitrile formic acid (B) in a gradient separation. Results were interpreted in comparison to the height of the intra‐run blank, with EFV levels >500 ng/mL and NVP levels >50 ng/mL found to be significant. The coefficient of variation ranged from 3% to 20% for EFV and 5% to 15% for NVP.

### 2.4 Data analysis

Descriptive analysis of EFV and NVP levels were performed, in relation to self‐reported maternal and infant ARV exposure, with median and interquartile ranges (IQR) calculated for each of the drugs tested at birth, 6‐, 10‐ and 14‐weeks. Analysis was performed using Microsoft Excel.

All mothers of infants enrolled in the study signed written informed consent for their infant's participation. This study was approved by the University of Pretoria's Faculty of Health Sciences Research Ethics Committee (Protocol number—41/2016).

## 3 Results

Of the 422 enrolled infants who were followed‐up at six weeks of age, 70 had sufficient left‐over plasma for NNRTI level testing at birth and 6‐weeks of age. Of these, 36 had samples at 10‐weeks and 35 at 14‐weeks of age (Table 1). There were 17 infants who had specimens available at all four time points.

**Table 1:** Maternal reported ARV exposure and feeding practice at the time of HIV PCR testing

		Birth	6 weeks	10 weeks	14 weeks
Number of infants; n		70	70	36	35
Infant body weight (kg); median (IQR)		3.1 (2.8 to 3.3)	4.5 (4.2 to 5.0)	5.7 (5.2 to 6.3)	5.9 (5.3 to 6.2)
Infant prophylaxis	Daily NVP; n (%)	69 (99%)	64 (91%)	12 (33%)	3 (9%)
	Daily NVP/AZT; n (%)	1 (1%)	0	0	0
	None; n (%)	0	0	24 (67%)	24 (68%)
	Unknown; n (%)	0	6 (9%)	0 (0%)	8 (23%)
Infant feeding	EBF; n (%)	68 (97%)	53 (76%)	29 (80%)	24 (69%)
	EFF; n (%)	2 (3%)	13 (18%)	1 (3%)	3 (8%)
	Mixed; n (%)	0	4 (6%)	0	0
	Unknown; n (%)	0	0	6 (17%)	8 (23%)
Mother taking cART	Yes; n (%)	70 (100%)	67 (96%)	26 (72%)	29 (74%)
	No; n (%)	0	3 (4%)	2 (6%)	3 (9%)
	Unknown; n (%)	0	0	8 (22%)	3 (17%)
Maternal cART regimen	EFV‐based; n (%)	66/70 (94%)	62/67 (93%)	25/26 (96%)	27/29 (93%)
	NVP‐based; n (%)	4/70 (6%)	5/67 (7%)	1/26 (4%)	2/29 (7%)
	Unknown; n (%)	0	0	0	0
Infant EFV plasma level (ng/mL)	Median (IQR)	1665 (1138 to 3045)	1227 (1094 to 3015)	1219 (909 to 1387)	0 (0 to 0)
Infant NVP plasma level (ng/mL)	Median (IQR)	3699 (2085 to 6043)	6888 (4020 to 11549)	3401 (2021 to 5123)	245 (101 to 307)

AZT, zidovudine; EBF, exclusive breastfeeding; EFF, exclusive formula feeding; EFV, efavirenz; NVP, nevirapine.

At time of delivery all 70 mothers were on a cART regimen, 66/70 (94%) on an EFV‐based regimen and 4/70 (6%) on a NVP‐based regimen. Median maternal age at time of delivery was 31 years (IQR: 28 to 36), with median time on cART of 170 days (IQR: 128 to 847). Two mothers were on treatment for <4‐weeks duration prior to delivery. The median birth weight was 3.1 kg (IQR: 2.8 to 3.3), with only five infants having a low birth weight of <2.5 kg.

**Figure 1:**
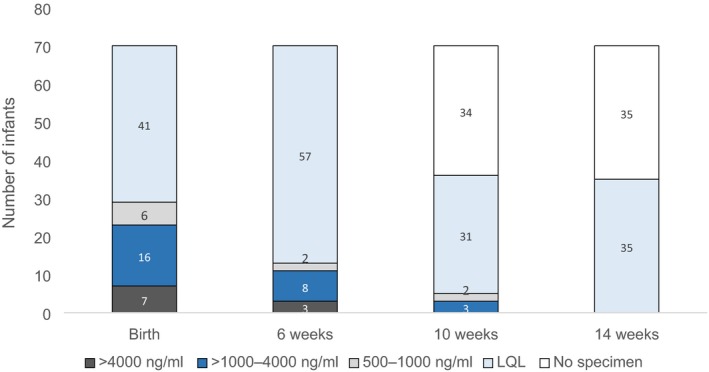
EFV levels in infants at time of HIV PCR testing. LQL, lower than quantification limit.

Figure 1 represents EFV levels of all 70 infants tested at birth and 6‐weeks of age, as well as those tested at 10‐ and 14‐weeks of age. At the time of HIV PCR testing at birth, of the 66 infants exposed to maternal EFV *in utero*, 29 (44%) had an EFV level >500 ng/mL: six (9%) had levels between 500 and <1000 ng/mL, 16 (24%) had EFV levels between 1000 and <4000 ng/mL and seven (11%) had levels ≥4000 ng/mL (Figure 1). Neither of the two infants born to women on an EFV‐based cART regimen for <4‐weeks at time of delivery (initiated seven and twelve days prior) had measurable EFV at birth. Among the five infants with a recorded low birth weight, four had an unmeasurable EFV level and one had a level of 1820 ng/mL (the mothers of these five infants had all been on EFV‐based cART for >4‐weeks at time of delivery). All women indicated that they were going to exclusively breastfeed except two, who indicated that they would exclusively formula feed. Infants born to both of these women had EFV levels ≥1000 ng/mL at time of birth testing. Only infants with mothers on an EFV containing regimen had a measurable EFV level.

At 6‐weeks of age, among 48 infants whose mothers were on an EFV‐based regimen and reported exclusive breastfeeding, 13 (27%) had an EFV level >500 ng/mL (11 had levels >1000 ng/mL). At 10‐weeks of age, among 25 infants whose mothers were on an EFV‐based regimen and reported exclusive breastfeeding, 5 (20%) had an EFV level >500 ng/mL (three had levels >1000 ng/mL). At 14 weeks of age, among 21 infants whose mothers were on an EFV‐based regimen and reported exclusive breastfeeding, none had a measurable EFV level (including two infants with detectable EFV levels at 10‐weeks who were tested again at 14‐weeks). None of the infants whose mothers reported being on an EFV‐containing regimen and were exclusively formula feeding or mixed feeding had measurable EFV levels at 6‐, 10‐ or 14‐weeks of age. Similarly, none of the infants whose mothers were not on an EFV‐based regimen or whose feeding history was unknown had a measurable EFV level.

**Figure 2:**
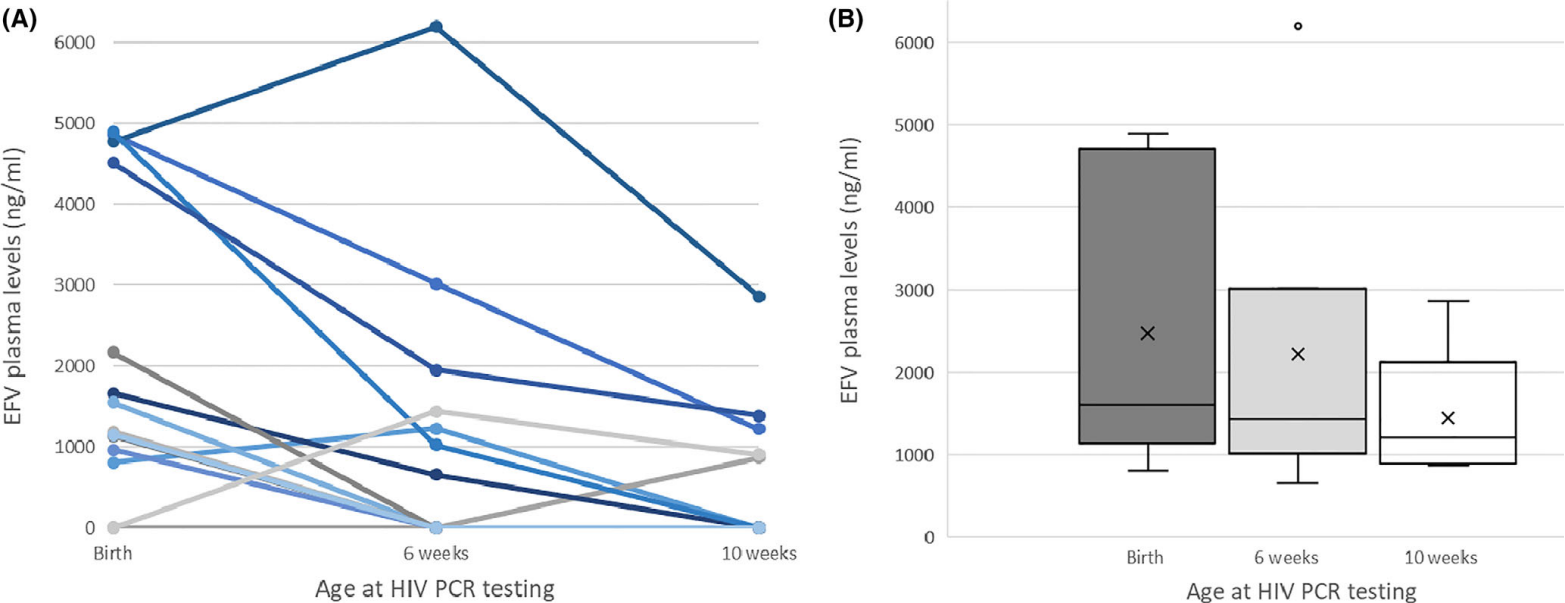
(**A**) Consecutive EFV levels among breastfed infants born to women on EFV‐based cART (n = 14) (**B**) EFV concentrations (medians with inter‐quartile ranges) among infants with a measurable level at birth (12/14), six‐weeks (7/14), and 10‐weeks (5/14).

Among the 37 infants who had consecutive samples available for ARV drug level testing at birth, 6‐weeks and 10‐weeks of age, 14 had been exposed to EFV *in utero* for >4 weeks and were exclusively breastfed. Among these infants, EFV levels of >500 ng/mL were found in 12/14 (86%) infants at birth, 7/14 (50%) at 6‐weeks of age and 5/14 (36%) at 10‐weeks of age (Figure 2A). Only 9/14 infants had NNRTI levels tested at 14‐weeks of age, none of whom had a measurable EFV level of >500 ng/mL (although only two of the infants with a measurable EFV level at 10‐weeks of age were tested at 14‐weeks). The median EFV level (among those with a measurable level) declined from 1607 ng/mL (IQR: 1146 to 4580) at time of birth testing, to 1436 ng/mL (IQR: 1022 to 2483) at 6‐week testing, and 1219 ng/mL (IQR: 909 to 1387) at 10‐week testing (Figure 2B).

**Figure 3:**
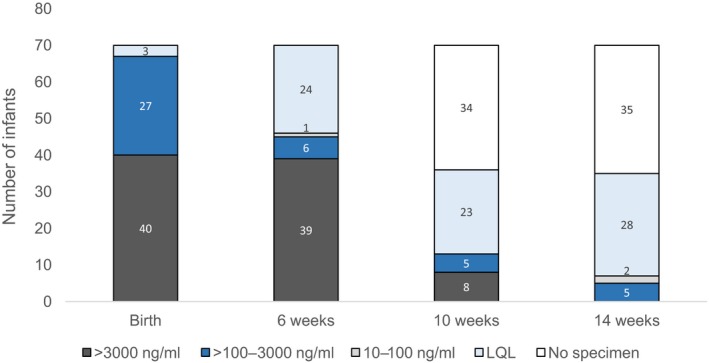
NVP levels in infants at time of HIV PCR testing. LQL, lower than quantification limit.

All infants were prescribed daily NVP prophylaxis from birth for at least a 6‐week duration. Age at first NVP dose occurred at a median of one hour postdelivery (IQR: 0 to 5) with age at blood sampling for HIV PCR birth testing occurring at a median of 25 hours (IQR: 9 to 38) postdelivery. Time from first infant NVP dose to blood sampling occurred at a median interval of 19 hours (IQR: 6 to 37). Among the 70 birth samples the median NVP level was 3699 ng/mL (IQR: 2046 to 6400): 67 (96%) infants had levels above the prophylactic trough concentration target of 100 ng/mL and 40 (57%) had levels above the adult therapeutic trough concentration target of 3000 ng/mL (Figure 3). One of the three infants with no detectable NVP received their first NVP dose post PCR blood sampling and the other two at 25 and 43 hours prior to sampling. Among the 4/70 infants born to women on a NVP containing regimen, all four had high NVP levels of 3967, 8068, 8286 and 16300 ng/mL.

At time of 6‐week testing, 64 infants were reported to still be taking daily NVP, of which 45 (70%) had NVP levels >100 ng/mL. Among these 64 infants, age at 6‐week testing took place at a median of 44 days (IQR: 42 to 46). At time of 10‐week HIV PCR testing, 36 infants were tested for NNRTI levels of which 12 were still taking daily NVP prophylaxis (as reported at maternal interview). The extended use of NVP syrup may have been because these infants were high‐risk, as defined by national guidelines. Thirteen (36%) infants had detectable NVP levels at 10‐weeks, all of which were >100 ng/mL. The one infant with a detectable NVP level (1678 ng/mL) who was not taking daily NVP prophylaxis at the time was being breastfed by a mother taking a NVP‐based regimen (Table 1). Hence, among the 23 infants at 10‐weeks of age who were not exposed to NVP at this time point, none had a measurable NVP level. At time of 14‐week HIV PCR testing, 35 infants were tested for NNRTI levels of which 3 were still taking daily NVP prophylaxis (as reported at maternal interview). Seven (20%) infants had a detectable NVP level of which five had a level >100 ng/mL. Among these five infants, three were still taking NVP prophylaxis (their mother's were not taking a NVP‐based regimen) and clinical information was outstanding for the remaining two. Hence, all infants who were still taking NVP prophylaxis at 10‐ and 14‐weeks of age had levels >100 ng/mL.

## 4 Discussion

This study describes the degree of exposure to NNRTI drugs among HIV‐exposed infants over the first few months of life. Over a third (35%) of newborn infants, whose mothers reported taking an EFV‐based regimen, had EFV levels equivalent to or above the recommended lower adult mid‐dosing treatment level at time of HIV PCR birth testing (i.e. >1000 ng/mL). Of these, 30% (7/23) had potentially toxic levels of >4000 ng/mL. Importantly, EFV is not approved for use in neonates on account of lack of safety and dosing data 24. The high EFV levels at birth are consistent with previous reports that foetuses are likely exposed to virologically suppressive drug concentrations *in utero* 25, 26, with pharmacogenomic heterogeneity possibly accounting for some of the variability (other than treatment adherence). Single nucleotide polymorphisms (SNPs) of the cytochrome P450 family of enzymes are known to influence plasma EFV concentrations. In particular, the *CYP2B6* 516G>T genotype has been described as a principal risk factor for toxicity‐related EFV levels in adults and has been found to confer relatively high plasma EFV levels in nursing infants 27, 28. Genotypic frequency studies conducted in black South African populations have described homozygous *CYP2B6* 516G>T SNP rates comparable with the prevalence in other African populations of around 12.5% 27, suggesting a non‐negligible proportion of South Africans will be poor metabolizers of both EFV and NVP 29. It is therefore possible that the 11% of newborn infants in this study that had EFV levels >4000 ng/mL represent such a population, especially considering that time of maternal EFV dosing and infant feeding are not thought to have a significant bearing on infant EFV plasma levels 28.

Among breastfed infants who had consecutive drug level testing, there was an overall decline in EFV levels over the first 10‐weeks postdelivery, likely a result of infant weight gain and induction of *CYP* enzymes. This finding is in keeping with previous studies evaluating EFV levels among breastfed infants whose mothers reported taking EFV‐based cART 26, 28, 30, 31. Among a cohort in Uganda, Gandhi and colleagues reported a mean plasma EFV concentration of 1694 ng/mL at birth which declined to 309 ng/mL at eight‐weeks and 297 ng/mL at 12‐weeks of age 26. In other studies, a median EFV level of 347 and 860 ng/mL has been reported among breastfed infants aged one month and 6 to 25 weeks of age respectively 30, 31. Interestingly, we found that among the four infants with plasma levels >4000 ng/mL at birth, all four maintained virologically suppressive levels at time of testing at 6‐weeks and three of the four still had EFV levels >1000 ng/mL at 10‐weeks of age (Figure 2A).

It is important to note that the minimum effective concentration of EFV for infants is unknown and that the described EFV mid‐dosing interval therapeutic range of between 1000 and 4000 ng/mL is derived from adult studies 21. Indeed, even among adults, the minimum effective concentration is uncertain where a range of between 470 and 760 ng/mL has been suggested 32. Bienczak and colleagues have proposed a therapeutic cut off of 650 ng/mL for infants, although the majority of infants below this threshold were still found to have viral load results <100 copies/mL 20. Hence, it is possible that as many as half of the HIV‐exposed infants tested in this cohort had virologically suppressive levels of EFV at birth and a third maintained therapeutic values at the time of 6‐week testing.

With regard to other NNRTI exposure, in addition to *in utero* and postnatal exposure to maternal EFV or NVP, all infants were prescribed daily NVP for at least a 6‐week duration. At the time of birth testing, all but three infants had NVP levels above the minimum prophylactic target of >100 ng/mL and more than half had levels above the standard therapeutic trough concentration target of 3000 ng/mL (albeit measured at a median of 17‐hours postdose). Detectable NVP levels at birth can be accounted for by workflow practices whereby NVP is routinely administered by nursing staff in labour ward immediately after delivery but infant blood is usually taken at a later time‐point by medical staff during routine working hours. Regarding the minimum effective concentration of NVP predictive of virological suppression among infants, it is important to note that it has not been possible to define a meaningful cut‐off ‐ possibly due to the combined effect of NRTI exposure as well as other variables including pre‐treatment viral load 22. Furthermore, it is unclear what effect, if any, simultaneous exposure to EFV and NVP have on NNRTI dose‐response.

The percentage of infants on NVP prophylaxis who maintain therapeutic levels of NVP at 6‐weeks of age is unknown. A population pharmacokinetic model has suggested that NVP prophylactic dosing of 15 mg once daily for infants >2.5 kg, as was provided to infants in this cohort, is likely to maintain therapeutic NVP levels for approximately a quarter of infants during the first 2‐weeks of life 23. However, the effect of host genetic polymorphisms, including the *CYP2B6* 516G>T genotype, was not taken into account and these estimates are therefore likely to be conservative. In two separate studies investigating NVP levels among infants receiving daily NVP prophylaxis of 4 mg/kg, median trough concentrations of 810 ng/mL at 4‐weeks of age among formula‐fed infants and >1000 ng/mL at 8‐weeks of age (maintained up to 6‐months of age) among breastfed infants have been reported 33, 34. This suggests suppressive levels of NVP at 6‐weeks of age are a possibility. Unfortunately, as timing of infant NVP dosing in relation to PCR testing was not recorded for this cohort (other than at birth), the proportion of infants who maintained virologically suppressive drug concentrations at 6‐weeks cannot be determined. However, the proportion of infants who had plasma levels of >100 ng/mL at the time of their 6‐week PCR testing may be used as a random marker of adherence to infant prophylaxis. Among infants whose mothers reported providing daily infant NVP syrup at time of their 6‐week HIV PCR test, only 70% had NVP levels >100 ng/mL. This suggests additional support to caregivers regarding adherence to infant prophylaxis may assist with further reducing the mother‐to‐child transmission rate.

A number of important limitations need to be considered regarding these findings. Testing was performed on a convenience sample of HIV‐exposed infants who were enrolled in a cohort study at a single facility. Hence, the results may not be generalizable on account of selection bias. Non‐nucleoside reverse transcriptase inhibitor trough levels at the various time points of testing would have provided more informative data, especially regarding NVP levels among infants taking prophylactic doses. Furthermore, maternal ARV testing at the same time as infant testing would have provided the extent of maternal treatment compliance within this cohort. Without this data it is not clear whether infants with no measurable EFV at birth (and whose mothers were receiving EFV during pregnancy) resulted from poor maternal treatment adherence or pharmacokinetic variability. Although signs of drug toxicity were not observed on clinical examination at birth, six‐, ten‐ and fourteen‐weeks of age, long‐term follow‐up will be required to rule‐out clinical sequelae (such as neuro‐developmental delay) secondary to high NNRTI levels during the neonatal and early infancy periods. As pharmacogenomic studies were not performed it was not possible to determine the association between certain clinical variables and NNRTI levels. Furthermore, NRTI drugs such as lamivudine and emtricitabine, which are also known to cross the placenta as well as be transferred in breastmilk, were not tested for thereby precluding a description of total ARV exposure in infants during the first months of life.

## 5 Conclusions

In summary, we describe virologically suppressive plasma levels of EFV, including potentially toxic levels, at the time‐points of EID testing among breastfed infants. These findings highlight the importance of careful monitoring of ARV side effects among HIV‐exposed infants and support recommendations that repeat HIV PCR testing be performed among all infants who test negative during the first months of life, especially considering time to virological rebound may be variable. Additionally, as only 70% of infants could be confirmed as being adherent to daily NVP prophylaxis at 6‐weeks of age, enhanced support to caregivers regarding adherence to infant prophylaxis needs to be considered as a means of further reducing the mother‐to‐child transmission rate.

## Competing interests

The authors declare that they have no competing interests.

## Authors’ contributions

AHM, GGS, TA, AEG, NdP contributed to study design. AHM, TYM and NdP involved in data collection. TS performed LCMS experiments. AHM, TYM, GGS, TS, JG, TA, AEG, MSP and NdP involved in data interpretation and analysis. AHM contributed to writing draft article. AHM, TYM, GGS, TS, JG, TA, AEG, MSP and NdP critically reviewed and approved final manuscript.
